# La_*n*(*n*+1)+*x*_Ni_*n*(*n*+5)+*y*_Si_(*n*+1)(*n*+2)–*z*_: A Symmetric Mirror Homologous Series in the La–Ni–Si System

**DOI:** 10.1021/acs.inorgchem.3c01194

**Published:** 2023-06-26

**Authors:** Davide Grilli, Volodymyr Smetana, Sheikh J. Ahmed, Vitalii Shtender, Marcella Pani, Pietro Manfrinetti, Anja-Verena Mudring

**Affiliations:** † Department of Materials and Environmental Chemistry, Stockholm University, Svante Arrhenius väg 16 C, Stockholm 10691, Sweden; ‡ DCCI, Department of Chemistry and Industrial Chemistry, University of Genova, Via Dodecaneso 31, Genova I-16146, Italy; § Institute SPIN-CNR, Corso Perrone 24, Genova I-16152, Italy; ∥ Department of Chemistry—Ångström Laboratory, Uppsala University, Box 538, Uppsala 75121, Sweden; ⊥ Department of Biological and Chemical Engineering and iNANO, Aarhus C 8000, Denmark

## Abstract

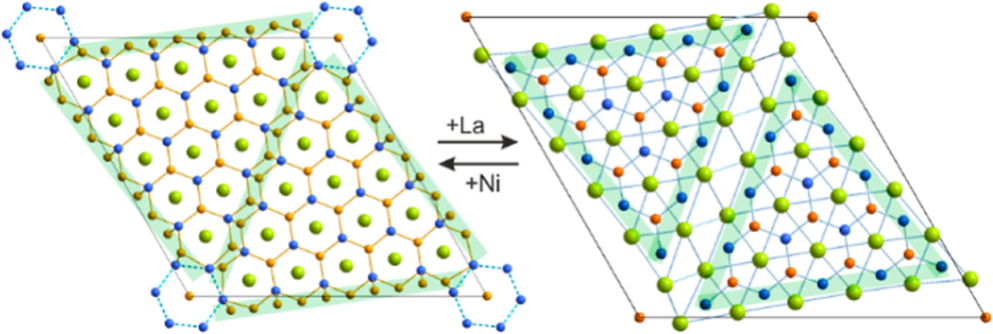

A series of four homologous silicides have been discovered during systematic explorations in the central part of the La–Ni–Si system at 1000 °C. All compounds La_12.5_Ni_28.0_Si_18.3_ (*n* = 3; *a* = 28.8686(8), *c* = 4.0737(2) Å, *Z* = 3), La_22.1_Ni_39.0_Si_27.8_ (*n* = 4; *a* = 20.9340(6), *c* = 4.1245(2) Å, *Z* = 1), La_32.9_Ni_49.8_Si_39.3_ (*n* = 5; *a* = 24.946(1), *c* = 4.1471(5) Å, *Z* = 1), and La_44.8_Ni_66.1_Si_53.4_ (*n* = 6; *a* = 28.995(5), *c* = 4.158(1) Å, *Z* = 1) crystallize in the hexagonal space group *P*6_3_/*m* and can be generalized according to La_*n*(*n*+1)+*x*_Ni_*n*(*n*+5)+*y*_Si_(*n*+1)(*n*+2)–*z*_ with *n* = 3–6. Their crystal structures are based on AlB_2_-type building blocks, fused La-centered Ni_6_Si_6_ hexagonal prisms, yielding larger oligomeric equilateral domains with the edge size equal to *n*. The domains extend along the *c* axis and show checkered ordering of the cationic and anionic parts, while all their atoms are located on mirror planes. La_*n*(*n*+1)+*x*_Ni_*n*(*n*+5)+*y*_Si_(*n*+1)(*n*+2)–*z*_ can be considered as a mirror series to the La-rich La_(*n*+1)(*n*+2)_Ni_*n*(*n*–1)+2_Si_*n*(*n*+1)_, where an exchange of the formal cationic and anionic sites, i.e., La and Si, occurs. The La–Ni–Si system is the first system where two such analogous series have been observed.

## Abstract 2

A homologous series La_*n*(*n*+1)+*x*_Ni_*n*(*n*+5)+*y*_Si_(*n*+1)(*n*+2)−*z*_ has been observed in the La−Ni−Si representing a symmetric Ni-rich mirror to the La_(*n*+1)(*n*+2)_Ni_*n*(*n*−1)+2_Si_*n*(*n*+1)._

## Introduction

Metal silicides have been intensely studied resulting in a substantial number of compounds showing a large variability of compositions and structural diversity.^1^ As functional materials, metal silicides have found broad applications as thermoelectrics,^2^ catalysts,^3^ magnetocalorics,^4,5^ superconductors,^6,7^ in electronics,^8^ high-temperature coatings,^9^ hydrogen sorption,^10,11^ or ion-battery anodes,^12^ to name some. In particular, ternary silicides with rare-earth and transition metals show extensive compositional diversity.^13^ Although the R–Ni–Si systems have been intensively explored in the past, new compounds can still be discovered. Some examples are systems with Ce,^14^ Gd,^15,16^ and Dy.^17^ Recent reinvestigation of isothermal sections of these systems yielded more than 20 ternary compounds in each system and broad homogeneity ranges in both ternary and binary (pseudoternary) regions.

When previously explored, the La–Ni–Si system yielded 20 ternary compositions including solid solutions.^1^ Among them, LaNiSi^6^ and La_3_Ni_4_Si_4_^18^ exhibit superconducting behavior (with *T*_c_ = 1.2 and 1.0 K, respectively), while LaNi_5–*x*_Si_*x*_ has been investigated for hydrogen sorption^19^ and battery applications.^20^ For practically all reported compounds (except for La_21_Ni_10.49_Si_15_^21^), isostructural representatives in other silicide systems have been reported, which may be the result of a rather focused research exploring isocompositional series and not investigating the whole phase space systematically. From a structural point of view, the La–Ni–Si system features a series of closely related compounds. The most prominent examples are the La_(*n*+1)(*n*+2)_Ni_*n*(*n*–1)+2_Si_*n*(*n*+1)_ series with four representatives (*n* = 2–5)^21^ and the extended NaZn_13_-related^22^ solid solution with multiple structural and compositional variations of La(Ni,Si)_12–13_.^23−25^

Our recent exploration of the phase space yielded four new compounds—LaNi_2_Si, La_3_Ni_3_Si_2_, La_6_Ni_7_Si_4_, and La_2_Ni_3_Si_2_,^26^ where the first one does not have an analogous known compound within the family of silicides, while the last one crystallized in its own structure type. La_3_Ni_3_Si_2_ and La_6_Ni_7_Si_4_ also show significant structural relationships with identical stacking motifs.^26^ Such a high density of compositions just in a narrow region (25–35 at. % La, 37–50 at. % Ni, and 24–29 at. % Si) motivated us for further explorations of the phase space. Just a little increase of the Si content to slightly above 30 at. % led to a series of closely related compounds with identical building principles. All these compounds strongly resemble those belonging to the already known series La_(*n*+1)(*n*+2)_Ni_*n*(*n*–1)+2_Si_*n*(*n*+1)_^21^ up to the same space group and building principles, however, with the clear exchange of the formal cationic and anionic sites, i.e., La and Si. In this work, we present detailed structural characterization of this mirror series which can generally be represented by the formula La_*n*(*n*+1)+*x*_Ni_*n*(*n*+5)+*y*_Si_(*n*+1) (*n*+2)–*z*_.

## Experimental Section

### Synthesis

All samples were prepared starting from pure elements purchased from Alfa Aesar—La slices (99.9 wt %) handled in a glove-box under Argon, Ni pieces (99.99 wt %), and Si grains (99.999 wt %). Compacted samples with a total mass of about 2 g each, were arc melted. The melting procedure was performed in an arc furnace with a thoriated tungsten electrode, on a copper hearth, and under a zirconium-gettered argon atmosphere. The buttons were remelted at least three times after turning them upside-down to ensure good homogenization. Negligible mass losses of less than 1% were encountered after arc melting. The as-cast samples were wrapped in Mo outgassed foil, closed in evacuated fused silica tubes, and annealed at 1000 °C for 7 days. At the end of the heat treatment, they were cooled to room temperature by switching off the furnace.

### Characterization

#### Powder X-ray Diffraction

Intensity data for powder X-ray diffraction (PXRD) analysis were collected using a BRUKER D2 2nd generation powder diffractometer [Bragg–Brentano geometry, Ni-filtered Cu K_α1_ radiation (λ = 1.5406 Å)] in 5–90° 2ϑ range, with 0.02° 2ϑ steps and total measurement time 30–60 min. For precise determination of lattice parameters, silicon powder was added as an internal standard to the sample powders. The sample purity has been analyzed with WinXPow software (Stoe&Cie, Darmstadt, Germany) by matching the measured and simulated powder patterns and, consequently, by Rietveld refinement (Figure S1, Table S1) with TOPAS software.^27^

#### Single Crystal X-ray Diffraction

Single-crystal X-ray diffraction (SCXRD) measurements were performed at room temperature on a Bruker D8 Venture diffractometer operating at 50 kV and 1.4 mA equipped with a Photon 3 CMOS detector, a flat graphite monochromator, and a Mo Kα IμS 3.0 microfocus source (λ = 0.71073 Å). The raw frame data were collected using the Bruker APEX3 software package (Bruker AXS, 2015), while the frames were integrated with the Bruker SAINT program using a narrow-frame algorithm for the integration of the data; absorption effects were corrected using the multi-scan method (SADABS).^28^ Initial models of the crystal structures were obtained with the program SHELXT-2014^29^ and refined using the program SHELXL-2014^30^ within the APEX3 software package. The atomic displacement parameters were refined anisotropically for all atoms. Diamond (Crystal Impact Gmbh, 2015) was used for the structure visualization.

#### Microscopy Analysis

Compositional analysis was performed with a JEOL 7000F scanning electron microscopy system equipped with Oxford Inca X-sight energy dispersive spectrometer at an acceleration voltage of 20 kV. The samples were mounted on a Bakelite support and finely polished prior to the analysis. Microscopic examinations were carried out in the back scattered electron mode. For elemental analysis, the La L alpha, Si K alpha, and Ni K-alpha edges were used. Energy-dispersive system optimization was done with a pure copper metal piece. Results of EDX analysis are presented in Figures S2–S5.

## Results and Discussion

Exploring the central part of the La–Ni–Si system along 32(1) at. % Si, we discovered a compound, which, at first sight, appeared to be a member of the La_(*n*+1)(*n*+2)_Ni_*n*(*n*–1)+2_Si_*n*(*n*+1)_ series.^21^ However, a more detailed inspection revealed the compound is significantly Ni richer, and further investigations led to discovery of three more closely related representatives of a new series. All compounds crystallize hexagonally in the space group *P*6_3_/*m* and can be generalized according to La_*n*(*n*+1)+*x*_Ni_*n*(*n*+5)+*y*_Si_(*n*+1)(*n*+2)–*z*_—La_12.5_Ni_28.0_Si_18.3_ (*n* = 3; *a* = 28.8686(8), *c* = 4.0737(2) Å, *Z* = 3), La_22.1_Ni_39.0_Si_27.8_ (*n* = 4; *a* = 20.9340(6), *c* = 4.1245(2) Å, *Z* = 1), La_32.9_Ni_49.8_Si_39.3_ (*n* = 5; *a* = 24.946(1), *c* = 4.1471(5) Å, *Z* = 1), and La_44.8_Ni_66.1_Si_53.4_ (*n* = 6; *a* = 28.995(5), *c* = 4.158(1) Å, *Z* = 1). Single crystal data and relevant parameters of the intensity data collections and structure refinements as well as atomic coordinates are reported in Tables 1 and S2–S5.

**Table 1:** Crystallographic Data and Details of Structure Refinements for Compounds of Series La_*n*(*n*+1)+*x*_Ni_*n*(*n*+5)+*y*_Si_(*n*+1)(*n*+2)-z_ with *n* = 3–6^a^

compound	*n* = 3	*n* = 4	*n* = 5	*n* = 6
CCDC	2174188	2174189	2174190	2174191
formula	La_12.5_Ni_28.0_Si_18.3_	La_22.1_Ni_39.0_Si_27.8_	La_32.9_Ni_49.8_Si_39.3_	La_44.8_Ni_66.1_Si_53.4_
formula weight, g·mol^–1^	3894.38	6134.26	8592.58	11598.48
*T* (K)	293(2)	296(2)	293(2)	295(2)
crystal system	hexagonal	hexagonal	hexagonal	hexagonal
SG	*P*6_3_/*m*	*P*6_3_/*m*	*P*6_3_/*m*	*P*6_3_/*m*
*a*, Å	28.8686(8)	20.9340(6)	24.946(1)	28.995(5)
*c*, Å	4.0737(2)	4.1245(2)	4.1471(5)	4.158(1)
*V*, Å^3^	2940.2(2)	1565.3(1)	2235.1(4)	3027(1)
*Z*	3	1	1	1
density (g/cm^3^)	6.60	6.51	6.38	6.36
μ (mm–^1^)	26.9	26.6	26.0	25.9
F(000)	5258	2738	3818	5150
index ranges	–37 ≤ *h* ≤ 37	–30 ≤ *h* ≤ 29	–35 ≤ *h* ≤ 35	–41 ≤ *h* ≤ 41
	–37 ≤ *k* ≤ 36	–29 ≤ *k* ≤ 30	–33 ≤ *k* ≤ 35	–35 ≤ *k* ≤ 38
	–5 ≤ *l* ≤ 5	–5 ≤ *l* ≤ 5	–5 ≤ *l* ≤ 5	–5 ≤ *l* ≤ 5
measured reflections	29995	24754	27671	30319
unique reflections	2329	1802	2532	3422
observed reflections	1857	1571	2158	2778
number of parameters	215	135	152	201
R_int_, R_sigma_	0.0626, 0.0334	0.0481, 0.0209	0.0392, 0.0197	0.0397, 0.0237
R, *wR* (observed)	0.0333, 0.0666	0.0334, 0.0652	0.0249, 0.0532	0.0247, 0.0518
R, *wR* (all data)	0.0563, 0.0735	0.0401, 0.0671	0.0323, 0.0562	0.0339, 0.0556
Δρ_max/min_ = (e·Å^–3^)	3.22/–2.33	1.53/–1.31	2.58/–2.21	1.86/–1.34

a and .

**Figure 1:**
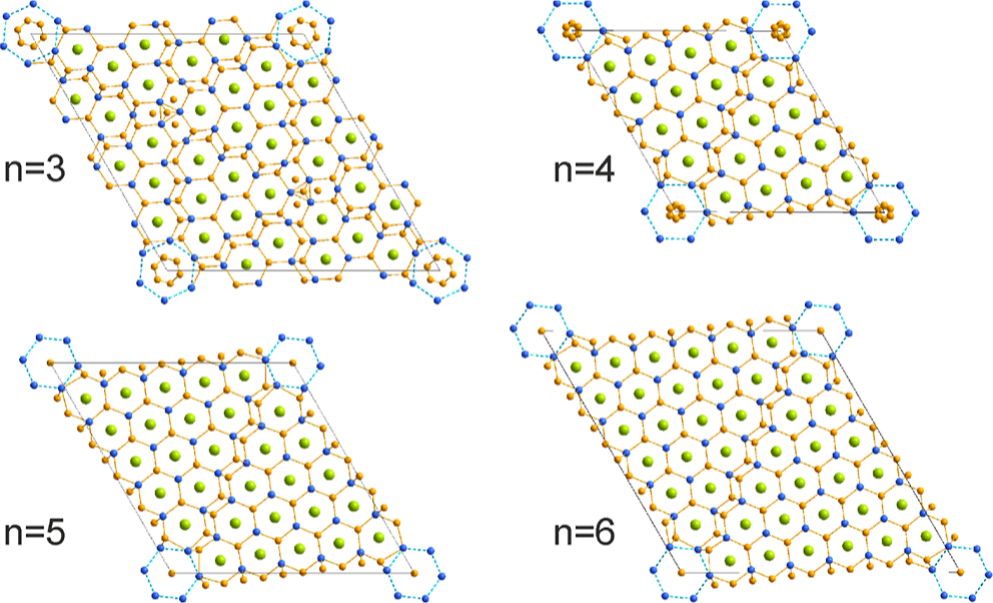
Projections of the idealized crystal structures of La_(*n*+1)(*n*+2)_Ni_*n*(*n*-1)+2_Si_*n*(*n*+1)_ with *n* = 3–6 on the corresponding *ab* planes. La atoms are colored green, Ni—orange, and Si—blue.

**Figure 2:**
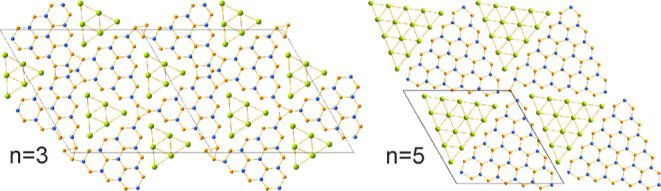
Idealized representation of a single layer in the crystal structures of La_*n*(*n*+1)+*x*_Ni_*n*(*n*+5)+*y*_Si_(*n*+1)(*n*+2)–*z*_ with *n* = 3 and 5. La atoms are colored in green, Ni in orange, and Si in blue.

The crystal structures of all compounds are best described based on ordered AlB_2_-type building blocks, as observed in SrPtSb.^31^ Here, they appear as fused La-centered Ni_6_Si_6_ hexagonal prisms (Figure 1). The prisms extend by sharing common bases along the *c* axis forming oligomeric equilateral triangular blocks parallel to the *ab* plane. The size of the blocks in this series varies from three to six of such fused prisms while, as a rule, each unit cell contains two blocks. In the case of *n* = 3, we observed a more complex arrangement with six blocks in the unit cell. The La and Ni/Si positions within a single block show clear separation forming alternating triangular groups at *z* = 0.25 or 0.75. These groups exchange between the neighboring blocks (Figure 2). Each block has 2*n*–1 common rhombic Ni_2_Si_2_ faces with identical neighboring blocks. The triangular blocks fit well with the concept of multicentered polyanionic clusters.^32,33^ In this light, we can distinguish two groups of Ni and Si atoms in terms of coordination—those from block faces and edges. In the first group, all Ni and Si atoms have triangular coordination Ni@Si_3_ or Si@Ni_3_ augmented by trigonal La_6_ prisms resulting in tricapped trigonal prisms. Those from the block edges are different—Ni@Si_4_ tetrahedra and Si@Ni_5_ pyramids complemented by La_4_ tetrahedra and La_5_ pyramids, respectively, resulting in conventional and bicapped tetragonal antiprism. Connectivity between the block edges occurs exclusively through Ni–Si bonds forming polyanionic corrugated facets with rhombic tiling (Figure S6). There is no direct connectivity between the block vertices. Moreover, their stacking results in substantial voids along the *c* axis leading to positional disorder.

The degree of the disorder varies between the compounds and is particularly the main feature of the structural diversity within the series. In *n* = 4, 5, and 6, we observe just one disordered region along the *c* axis (*x*, *y* ≈ 0–0.1). In contrast to the related pnictides,^34^ this disorder extends significantly beyond the unique transition metal site directly around the 6_3_ axis but still can easily be rationalized. Taking *n* = 5 as an example for illustration (Figure S7), the central part around the 6_3_ is covered by multiple Ni sites at (2*a*) and around the *c* axis at (6*h*) with the total occupation not exceeding one fully occupied 2*a* site. The disordered Ni “cluster” is surrounded by a triangle of La/Si split positions again approaching one fully occupied position (6*h*) where 55–60% belongs to a positionally ordered single Si site. The last wave of disorder includes partially occupied (SOF ≈ 80%) Ni positions. The latter Ni and Si participate in the formation of the above-mentioned hexagonal prisms, simultaneously serving as a boundary between the ordered and disordered parts of the structure. In general, within the La_*n*(*n*+1)+*x*_Ni_*n*(*n*+5)+*y*_Si_(*n*+1)(*n*+2)–*z*_ series, the deviation from a fixed composition, which is represented by the three coefficients *x*, *y*, and *z*, behaves somewhat differently in different cases. For *n* = 4–6, *x* is identical to *z* as all the disorder regarding La and Si is solely about their common split position. As a rule, this value varies between 2 and 3. *y* is though a more complex parameter reflecting heavy disorder around the *c* axis and minor occupational disorder in the triangular blocks. Here, we see that for *n* = 4, the total occupation of the tunnel space results in a high *y* that is also observed for *n* = 3, while for *n* = 5 and 6, both disordered regions compensate each other resulting in *y* ≈ 0.

The general disorder picture in *n* = 3 is slightly different and more complex moving toward partial ordering together with disorder differentiation. The channel centered at (0, 0, *z*) does not contain any positions directly at the *c* axis and is represented only by two Ni positions with SOF ≈ 25% (6*h* and 12*i*) and a La/Si split position with Si occupying 75%. The latter feature is common for the whole series, although no disordered positions have been observed beyond this line. The second channel centered at (2/3, 1/3, *z*) or at (1/3, 2/3, *z*) is located at the 3-fold axis and affects rather limited number of positions and shows certain differentiation along the *c* axis. This disorder is related to the alternation of the anionic and cationic triangular groups. Where anionic groups closely approach each other, their boundary Si positions get partially occupied and additional complementary Ni sites could be refined between them at (1/3 2/3 1/4). The cationic triangles are separated by a number of disordered Ni positions forming triangles around (1/3 2/3 3/4) or (2/3 1/3 1/4) that in total represent one fully occupied 6*h* position.

Having four compounds in the series raises a question whether more of them can still be discovered, particularly in the light that representatives with *n* = 1 and 2 are known for pnictides (Table 2). Additionally, we shall not forget about the related series La_(*n*+1)(*n*+2)_Ni_*n*(*n*–1)+2_Si_*n*(*n*+1)_ (*n* = 2–5).^21^ All our attempts to extend the series did not succeed, and the end members of the series (*n* = 3 and 6) have been found in equilibrium with other ternary compounds practically excluding further expansion. For instance, according to PXRD analysis, the area of the possible existence of *n* = 2 does not show any signs of related hexagonal phases or even the presence of *n* = 3 and this may have a reasonable explanation. The crystal structure of *n* = 3 does not exactly follow the trend and is in fact a superstructural variant, probably because of geometric reasons—a packing of smaller triangular blocks could not be achieved without substantial rearrangement of the disordered areas between them. Following this tendency, the possible structure of *n* = 2 would require even more significant rearrangements practically destroying the small triangular blocks.

**Table 2:** List of Homologous Lanthanide Compounds Based on a Phase-Sharing Trigonal Prism Architecture

Ni rich			
	R–Ni–Si	R–Ni–P	R–Ni–As
*n* = 1		Ni_2_P^35^	
*n* = 2		Sc_2_Ni_12_P_7,_^36^ R_2_Ni_12_P_7_ (R = Ho, Er)^37^	Eu_2_Pd_12_As_7_^36^ R_2_Co_12_As_7_ (R = Y, Ce–Yb)^38^
*n* = 3	La_12.5_Ni_28.0_Si_18.3_	Ho_6_Ni_20_P_13_^37^ R_7_Ni_19_P_13_ (R = Y, La–Sm, Gd–Tm)^39,40^	R_7_Ni_19_As_13_ (R = Y, La–Sm, Gd–Tm)^39,40^
*n* = 4	La_22.1_Ni_39.0_Si_27.8_	R_3_Ni_7_P_5_ (R = La–Sm, Gd, Tb)^41^ R_12_Ni_30_P_21_ (Y, Ce, Pr, Er)^42−45^ R_20_Ni_42_P_30_ (R = Y, Ce–Eu)^34,43,46−50^	R_6_Ni_15_As_10_ (R = Y, Sm, Gd–Dy)^51,52^ Er_13_Ni_25_As_19_^42^ R_20_Ni_42_As_30_ (R = Y, La–Dy)^40,46,53^
*n* = 5	La_32.9_Ni_49.8_Si_39.3_	R_15_Ni_28_P_21_ (R = Y, Sm, Tb)^45,47,50^	
*n* = 6	La_44.8_Ni_66.1_Si_53.4_		

R rich			
	La–Ni–Si	R–Ni–Si	R–T–Si
*n* = 2	La_6_Ni_2_Si_3_^21^	R_6_Ni_1.6+*x*_Si_3_ (R = Ce–Nd, Gd)^15,54−56^	R_6_Co_1.6+*x*_Si_3_ (R = Ce, Nd, Sm, Tb)^57−59^
*n* = 3	La_5_Ni_2_Si_3_^21^	R_5_Ni_2_Si_3_ (R = Ce, Pr)^60,61^	
*n* = 4	La_15_Ni_7_Si_10_^21^	Pr_15_Ni_7_Si_10_,^62^ Ce_15_Ni_4_Si_13_^63^	
*n* = 5	La_21_Ni_11_Si_15_^21^	Nd_42_Ni_22–*x*_Si_31_^56^	

**Figure 3:**
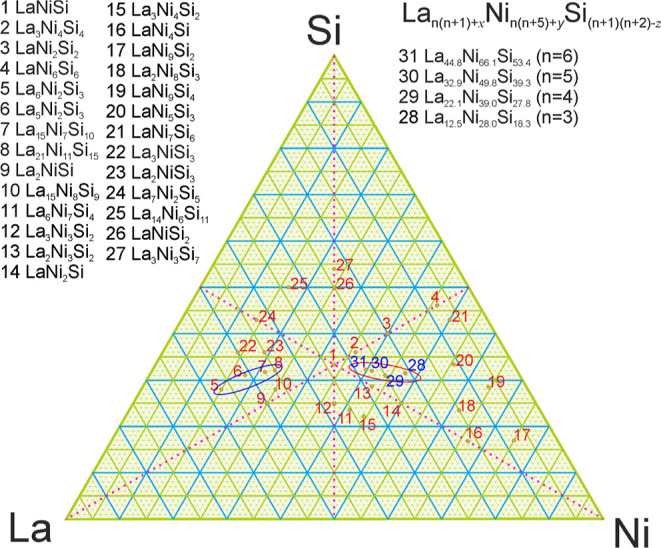
The map of reported and newly discovered ternary compounds in the La–Ni–Si system. Two homologous series are highlighted with blue and red ellipses, respectively.

On the other side, *n* = 6 was found in equilibrium with the known phase LaNiSi.^6^ Moreover, *n* = 6 has never been reported in any other system and La_44.8_Ni_66.1_Si_53.4_ is the only representative of such high order. A more precise look on the system reveals that both the La_(*n*+1)(*n*+2)_Ni_*n*(*n*–1)+2_Si_*n*(*n*+1)_^21^ and R_*n*(*n*+1)+*x*_Ni_*n*(*n*+5)+*y*_Si_(*n*+1)(*n*+2)-*z*_ series form almost straight lines aiming toward LaNiSi (Figure 3). Both series can be considered with some degree of approximation as compositional mirrors with respect to the LaNi–Si line, while LaNiSi itself can be considered as the compositional endpoint of both series, where *n* = ∞. Indeed, the crystal structure of LaNiSi contains many identical local motifs serving as an ultimate member unifying both homologous rows (Figure S8). However, the ZrBeSi type^64^ is the structural endpoint consisting of just one uniform building block—a centered heteroatomic hexagonal prism. In this light, the La–Ni–Si system is, so far, unique with respect to the reported isostructural and related homologous compounds. Interestingly, Ni-richer representatives of the series have only been observed and characterized solely with pnictides, while lanthanide richer ones—with silicides (Table 2). The only compound in this series without any *p* element, Y_9_Co_7–*x*_Pd_*x*_,^65^ and extension of the known series in the La–Ni–Si system indicate that such homologous rows may be even more common being controlled presumably by geometric reasons.

**Figure 4:**
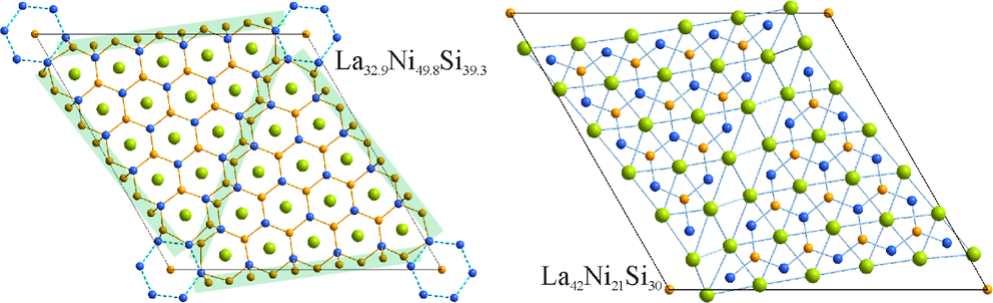
Comparison of the corresponding members of the R_*n*(*n*+1)+*x*_Ni_*n*(*n*+5)+*y*_Si_(*n*+1)(*n*+2)–*z*_ and La_(*n*+1)(*n*+2)_Ni_*n*(*n*–1)+2_Si_*n*(*n*+1)_ series with *n* = 5. La atoms are colored green, Ni atoms are colored orange, and Si atoms are colored blue.

What is the main difference between both series in the La–Ni–Si system? We will illustrate it using two respective silicides with *n* = 5—La_42_Ni_21_Si_30_^21^ and La_32.9_Ni_49.8_Si_39.3_. In addition to the formal exchange of the La and Si roles, we may also immediately notice a significant (more than double) increase in Ni fraction and, accordingly, the total number of atoms per unit cell. Such a significant change is not directly reflected in the unit cell volume, showing just a negligible increase of around 0.5% meaning higher packing efficiency (6.38 vs 5.91 g/cm^3^). Comparing both structures and taking into account La/Si exchange, it can easily be noticed that the only difference between them comes from Ni “walls” around basically identical but inverted triangular blocks (Figure 4, shaded areas). This area remains unoccupied in the La-richer representatives. In this light, it is interesting to inspect how La–La distances change between the series. In the Ni-richer La_32.9_Ni_49.8_Si_39.3_ with isolated cationic groups, all La–La distances are in a narrow range of 4.0763–4.1341(2) Å. Instead, La_42_Ni_21_Si_30_ shows a high variability of such distances: 3.621–4.154(2) Å. This variability is rather uniform within and between the triangular blocks. However, neighboring cationic triangular blocks are now directly connected, which is also expressed in the unit cell expansion along the *c* axis introducing one longer La–La contact—4.352(1) Å.

## Conclusions

A series of homologous silicides, La_*n*(*n*+1)+*x*_Ni_*n*(*n*+5)+*y*_Si_(*n*+1)(*n*+2)–*z*_ (*n* = 3–6), have been discovered during systematic explorations in the central part of the La–Ni–Si system at 1000 °C. SCXRD analysis revealed that all compounds crystallize in the hexagonal space group *P*6_3_/*m* and are based on identical building principles. The crystal structures of all compounds can be represented based on ZrBeSi type fragments condensing into bigger triangular blocks extending along the *c* axis. The newly discovered silicides belong to a larger family of compounds that was until recently clearly divided into two groups, structurally and compositionally. The pnictides were represented by transition metal-rich compositions, while silicides—rich by lanthanide, showed slightly different connectivities between the triangular blocks and packing efficiency. The La–Ni–Si system was found to be unique, simultaneously accommodating two homologous series and establishing some connectivity between the pnictides and silicides chemistry. The new compounds not only presented new connectivity regimes between the triangular blocks (*n* = 3) but also offered expansion of the triangular blocks themselves (up to *n* = 6). A quick inspection of the reported phase diagrams of the related R–Ni–Si systems, e.g., with Ce^14^ or Gd,^15^ indicated identical or closely related compounds may have eventually been detected but never characterized in detail suggesting a favorable direction for our research. In the light of the broad occurrence of the pnictide series with all lanthanides and certain influence of both chemical and geometric aspects, silicides appear as promising candidates, particularly for further expansion of the research area toward higher order block formations.

## Supporting Information Available

PXRD patterns, refined cell parameters, SEM–EDX images, Ni/Si rhombic tiling of the triangular block edges, analysis of the axial disorder in the crystal structure, [110] projection of the crystal structure, and atomic coordinates and equivalent thermal displacement parameters (PDF)
