# Supplementary material for: Lan(n+1)+xNin(n+5)+ySi(n+1)(n+2)–z: A Symmetric Mirror Homologous Series in the La–Ni–Si System
Source: Inorg Chem. 2023 Jun 26;62(27):10736–42. doi: 10.1021/acs.inorgchem.3c01194 (PMC10336971; doi:10.1021/acs.inorgchem.3c01194)
Supplement: Supplementary file 1 — ic3c01194_si_001.pdf [file ic3c01194_si_001.pdf]

Supporting Information

$La_{n(n+1)+x}Ni_{n(n+5)+y}Si_{(n+1)(n+2)-z}$: a symmetric mirror homologous series in the La-Ni-Si system

Davide Grilli,[a,b] Volodymyr Smetana,[a] Sheikh J. Ahmed,[a] Vitalii Shtender,[c] Marcella Pani*,[b], Pietro Manfrinetti*,[b], Anja-Verena Mudring*,[a,d]

[a]*Department of Materials and Environmental Chemistry, Stockholm University, 10691 Stockholm, Sweden*

[b]*DCCI, Department of Chemistry and Industrial Chemistry, University of Genova, I-16146, Genova, Italy & Institute SPIN-CNR, Genova, I-16152, Italy*

[c]*Department of Chemistry – Ångström Laboratory, Uppsala University, 75121 Uppsala, Sweden*

[d] *Department of Biological and Chemical Engineering and iNANO, Aarhus University, 8000 Aarhus C, Denmark.*

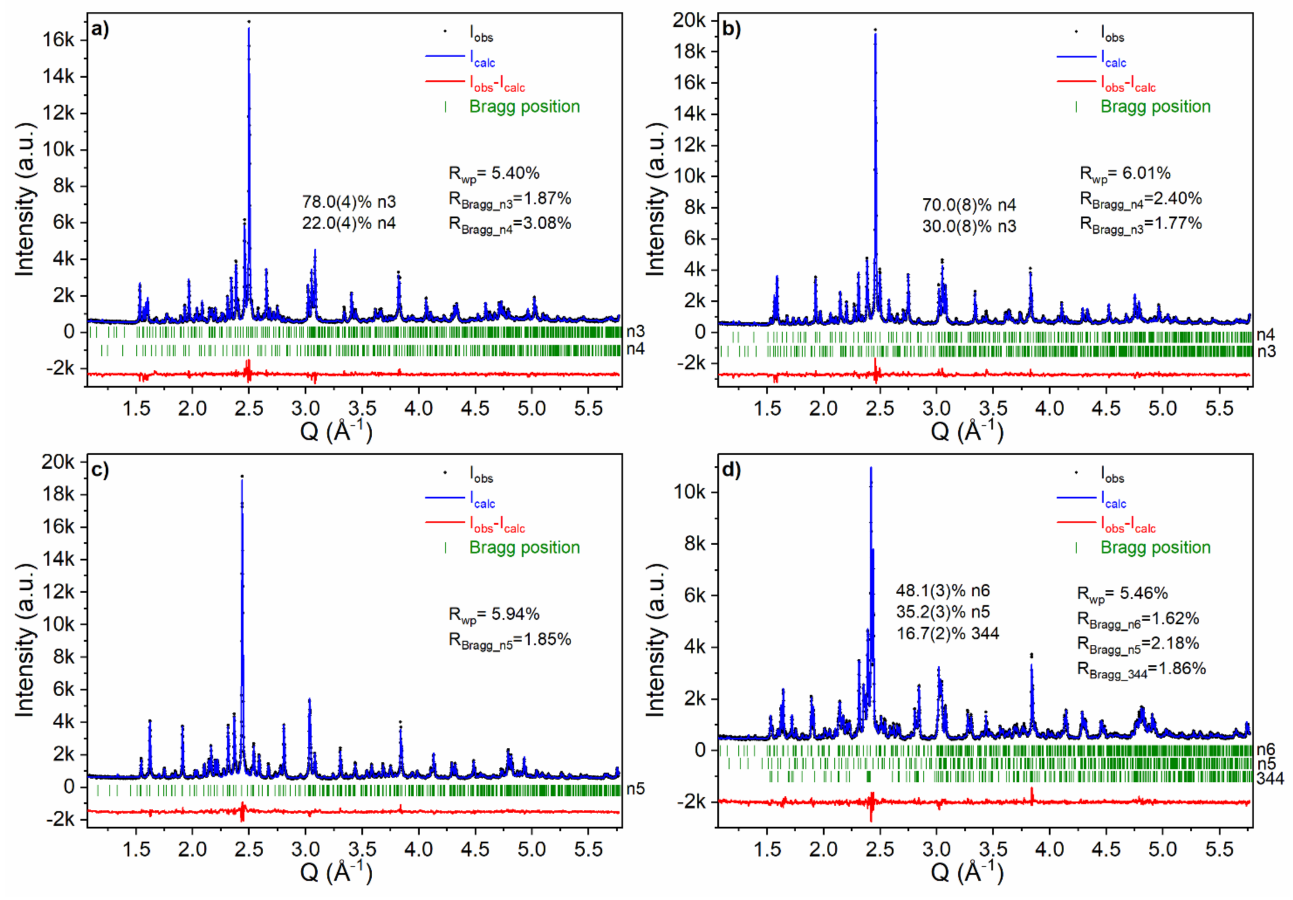

Figure S1. Measured PXRD patterns of the samples $La_{n(n+1)+x}Ni_{n(n+5)+y}Si_{(n+1)(n+2)-z}$ with n = 3-6.

Table S1. Refined cell parameters of $La_{n(n+1)+x}Ni_{n(n+5)+y}Si_{(n+1)(n+2)-z}$ with n = 3-6 as obtained from the samples from Figure S1.

sample	phase	cell parameters		
		a /Å	*c* /Å	*V* /Å^3
**n3**	n = 3	28.8537(4)	4.08112(6)	2942.48(9)
	n = 4	20.9688(5)	4.12256(12)	1569.80(8)
**n4**	n = 4	20.9632(3)	4.12301(8)	1569.13(5)
	n = 3	28.8315(16)	4.0851(3)	2940.8(4)
**n5**	n = 5	24.9493(4)	4.14656(7)	2235.29(8)
**n6**	n = 6	29.0001(4)	4.16526(7)	3033.69(11)
	n = 5	24.9503(6)	4.14896(12)	2236.77(12)

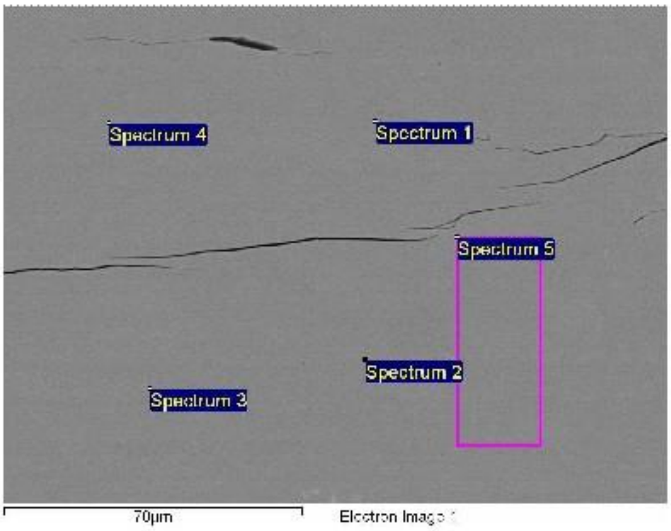

Spectrum	In stats.	Si	Ni	La
Spectrum 1	Yes	32.19	45.16	22.65
Spectrum 2	Yes	32.03	45.11	22.86
Spectrum 3	Yes	32.11	45.02	22.87
Spectrum 4	Yes	31.74	45.59	22.67
Spectrum 5	Yes	31.60	45.31	23.09

Figure S2. SEM-EDX image of compound n=3 shows homogeneity across the whole area of interest. The result compares well with the refined composition obtained from single crystal X-ray diffraction analysis, $La_{12.5}Ni_{28.0}$ $Si_{18.3}$ (at. %: La 21.3, Ni 47.6, Si 31.1).

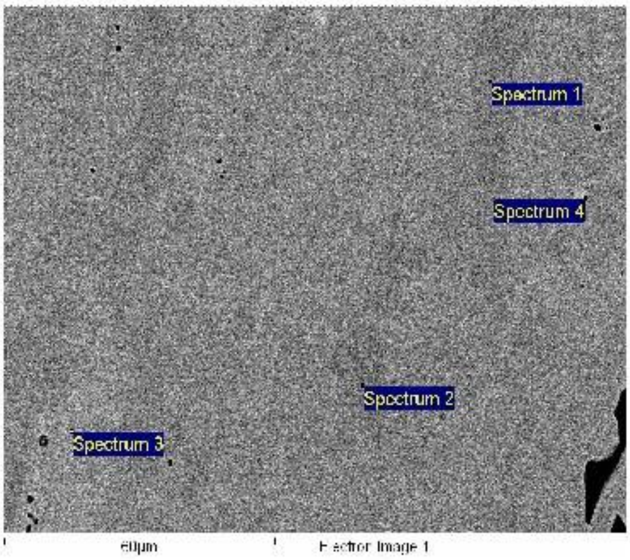

Spectrum	In stats.	Si	Ni	La
Spectrum 1	Yes	31.85	44.91	23.25
Spectrum 2	Yes	31.97	44.42	23.60
Spectrum 3	Yes	34.27	41.07	24.67
Spectrum 4	Yes	31.01	41.55	27.44

Figure S3. SEM-EDX image of compound n= 4 shows inhomogeneity across chosen region, which is marked by the variation in image contrast and also in the composition. Single crystal diffraction points towards a composition $La_{22.1}Ni_{39.0}$ $Si_{27.8}$ (at. %: La 24.8, Ni 43.9, Si 31.3).

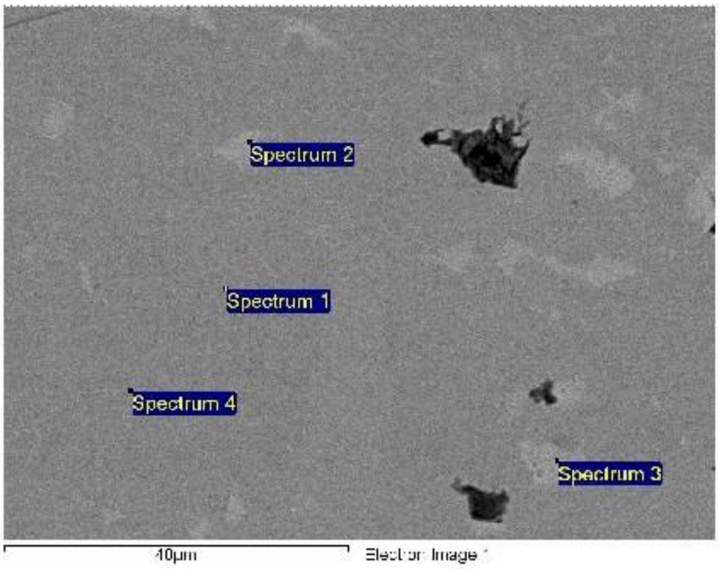

Spectrum	In stats.	Si	Ni	La
Spectrum 1	Yes	32.59	39.60	27.81
Spectrum 2	Yes	24.69	38.38	36.94
Spectrum 3	Yes	24.66	39.18	36.16
Spectrum 4	Yes	33.45	39.57	26.97

Figure S4. SEM-EDX image of $La_{n(n+1)+x}Ni_{n(n+5)+y}Si_{(n+1)(n+2)-z}$ with n=5 showing a multiphase sample. X-ray powder diffraction analysis showed this phase to be the major phase, with the composition refined by single crystal corresponding to $La_{32.9}Ni_{49.8}Si_{39.3}$ (at. %: La 27.0, Ni 40.8, Si 32.2).

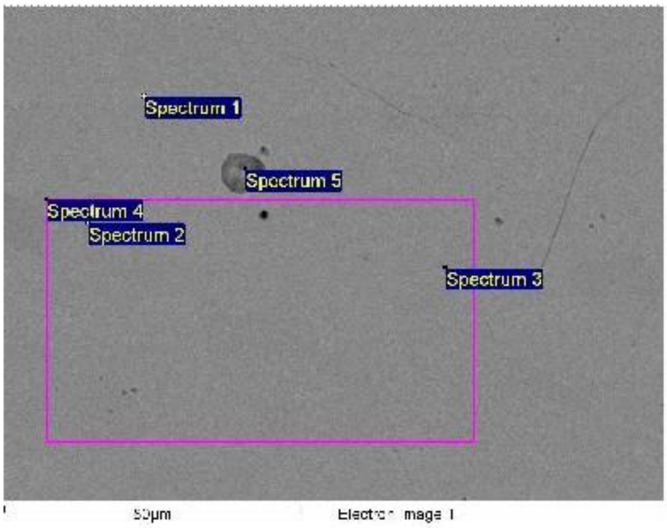

Spectrum	In stats.	Si	Ni	La
Spectrum 1	Yes	33.34	38.97	27.69
Spectrum 2	Yes	35.49	37.33	27.17
Spectrum 3	Yes	32.77	38.73	28.50
Spectrum 4	Yes	33.76	38.67	27.58
Spectrum 5	Yes	14.55	49.54	35.91

Figure S5. SEM-EDX image of $La_{n(n+1)+x}Ni_{n(n+5)+y}Si_{(n+1)(n+2)-z}$ with n=6 shows good agreement with single crystal data: refined composition $La_{44.8}Ni_{66.1}Si_{53.4}$ (at. %: La 27.3, Ni 40.2, Si 32.5).

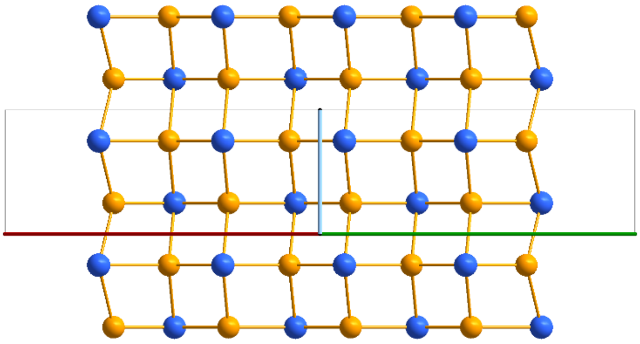

Figure S6. Ni/Si rhombic tiling of the triangular block edges. Ni atoms are colored orange and Si blue. Crystallographic axes are color-coded: *a* – red, *b* – green, *c* – blue.

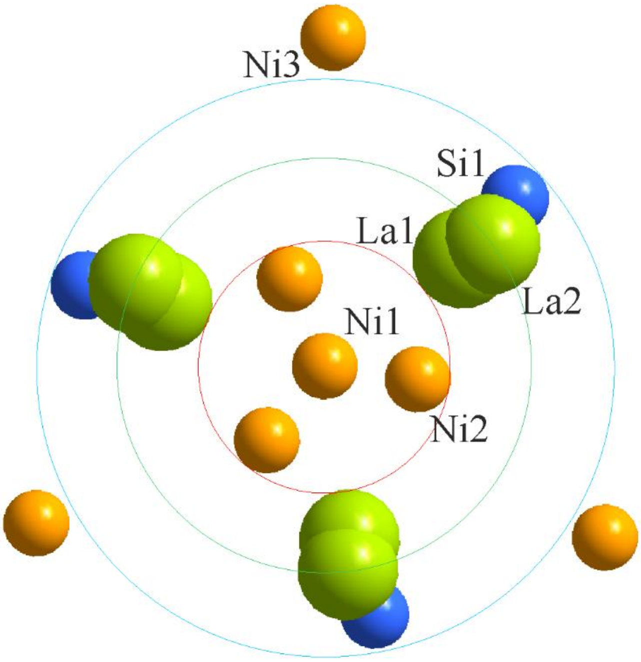

Site	*SOF*
Ni1 (2a)	*0.24(1)*
Ni2 (6h)	*0.12(2)*
Ni3 (6h)	*0.770(4)*
Si1 (6h)	*0.55(4)*
La1 (6h)	*0.17(2)*
La2 (12i)	*0.15(1)*

Figure S7. Analysis of the axial disorder in the crystal structure of $La_{n(n+1)+x}Ni_{n(n+5)+y}Si_{(n+1)(n+2)-z}$ with $n = 5$ at $z \approx$ ¼ or ¾. Position Ni1 here is located directly at $x = 0$, $y = 0$.

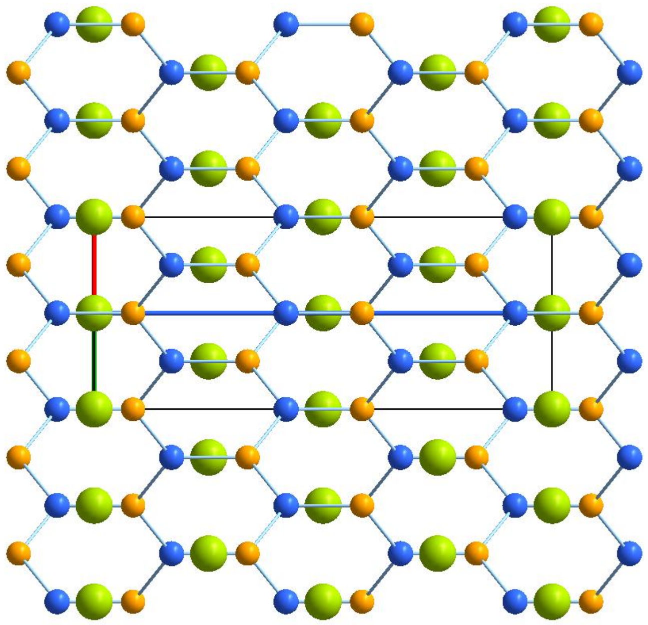

Figure S8. [110] projection of the crystal structure of LaNiSi. Crystallographic axes are color-coded: *a* – red, *b* – green, *c* – blue. La atoms are colored green, Ni orange, Si blue.

Table S2. Atomic coordinates and equivalent thermal displacement parameters for n = 3.

Site	*x*	*y*	*z*	U_{eq} /Å^2	SOF
La1	0.04668(2)	0.44175(2)	¾	0.0098(1)	1
La2	0.97446 (2)	0.27756(2)	¾	0.0102(1)	1
La3	0.13828(2)	0.37018(2)	¾	0.0096(1)	1
La4	0.88349 (2)	0.34983(2)	¾	0.0110(1)	1
La5	0.20526(2)	0.13825(2)	¼	0.0143(1)	1
La6	0.21584(2)	0.53956(3)	¾	0.0183(2)	1
La0A	0.05771(9)	0.0801(1)	¼	0.0184(9)	0.255(3)
Si0A	0.0732(2)	0.1261(2)	¼	0.0201(9)	0.745(3)
Ni0B	0.0878(3)	0.0597(3)	¼	0.020(3)	0.251(8)
Ni0C	0.0548(2)	0.0384(2)	0.113(1)	0.026(2)	0.230(5)
Ni1A	0.2786(1)	0.6626(1)	¾	0.046(3)	0.61(2)
Ni1B	0.2886(3)	0.6631(2)	0.491(4)	0.067(4)	0.239(9)
Ni1C	⅓	⅔	¼	0.031(2)	0.58(1)
Si1D	0.3295(3)	0.7118(3)	¼	0.029(3)	0.41(1)
Ni1	0.04059(5)	0.52321(5)	¼	0.0122(3)	1
Ni2	0.18919(5)	0.29535(5)	¾	0.0118(3)	1
Ni3	0.22620(5)	0.37719(5)	¼	0.0128(3)	1
Ni4	0.13249(5)	0.44926(5)	¼	0.0130(3)	1
Ni5	0.19762(5)	0.61002(5)	¼	0.0160(3)	1
Ni6	0.29422(5)	0.53413(5)	¼	0.0166(3)	1
Ni7	0.12038(6)	0.13580(7)	¾	0.0381(4)	1
Ni8	0.96651 (5)	0.35606(5)	¼	0.0125(3)	1
Ni9	0.12446(5)	0.56920(5)	¾	0.0127(3)	1
Ni10	0.05976(5)	0.28318(5)	¼	0.0125(3)	1
Ni11	0.26218(5)	0.46095(5)	¾	0.0135(3)	1
Ni12	0.15525(5)	0.21428(5)	¼	0.0134(3)	1
Si1	0.0529(1)	0.36273(9)	¼	0.0084(5)	1
Si2	0.2680(1)	0.3813(1)	¾	0.0112(6)	1
Si3	0.1242(1)	0.5267(1)	¼	0.0110(5)	1
Si4	0.0384(1)	0.5619(1)	¾	0.0109(5)	1
Si5	0.1971(1)	0.2176(1)	¾	0.0128(6)	1
Si6	0.2171(1)	0.4541(1)	¼	0.0109(5)	1
Si7	0.1472(1)	0.2918(1)	¼	0.0115(5)	1
Si8	0.2033(1)	0.6565(1)	¾	0.0250(7)	1

Table S3. Atomic coordinates and equivalent thermal displacement parameters for n = 4.

Site	*x*	*y*	*z*	U_{eq} /Å^2	SOF
La1	0.50657(2)	0.62826(2)	¾	0.0089(1)	1
La2	0.29515(2)	0.45501(2)	¾	0.0095(1)	1
La3	⅓	⅔	¾	0.0098(2)	1
La4	0.27891(3)	0.19713(3)	¼	0.0132(1)	1
La0A	0.096(2)	0.111(2)	0.68(2)	0.041(8)	0.07(2)
Ni1	0.34137(6)	0.36818(6)	¼	0.0111(2)	1
Ni2	0.44582(6)	0.45661(6)	¾	0.0120(2)	1
Ni3	0.23538(6)	0.28467(6)	¾	0.0133(2)	1
Ni4	0.40610(6)	0.24143(6)	¾	0.0120(2)	1
Ni5	0.37904(6)	0.58186(6)	¼	0.0114(2)	1
Ni6A	0.1430(4)	0.2020(5)	¼	0.025(2)	0.555(8)
Ni6B	0.1260(4)	0.1994(6)	0.309(3)	0.017(2)	0.2
La7A	0.086(1)	0.1047(8)	¾	0.019(4)	0.21(4)
Si7B	0.1478(3)	0.1498(2)	¾	0.021(2)	0.63(2)
Ni8A	0.0243(3)	0.0763(4)	0.620(2)	0.027(2)	0.170(5)
Ni8B	0.002(3)	0.039(3)	¾	0.027(2)	0.034(4)
Ni8C	0.0332(4)	0.1156(6)	¾	0.021(3)	0.163(8)
Si1	0.4630(1)	0.7120(1)	¼	0.0066(4)	1
Si2	0.4250(1)	0.4973(1)	¼	0.0091(4)	1
Si3	0.3634(1)	0.3281(1)	¾	0.0104(4)	1
Si4	0.2135(1)	0.3244(1)	¼	0.0126(4)	1

Table S4. Atomic coordinates and equivalent thermal displacement parameters for n = 5.

Site	*x*	*y*	*z*	U_{eq} /Å^2	SOF
La1	0.44215(2)	0.70767(2)	¼	0.00999(7)	1
La2	0.46881(2)	0.88336(2)	¼	0.00879(7)	1
La3	0.31920(2)	0.90966(2)	¼	0.00866(7)	1
La4	0.61837(2)	0.85674(2)	¼	0.00928(7)	1
La5	0.17004(2)	0.93889(2)	¼	0.01225(7)	1
La6A	0.0854(9)	0.0151(5)	¾	0.017(2)	0.17(2)
Si6B	0.1206(6)	0.0144(5)	¾	0.033(3)	0.55(4)
La6C	0.1012(7)	0.0208(4)	0.805(4)	0.027(2)	0.15(1)
Ni1	0.16789(5)	0.05592(6)	¼	0.0290(4)	0.770(4)
Ni2	0.36052(3)	0.84426(3)	¾	0.0112(1)	1
Ni3	⅓	⅔	¾	0.0113(2)	1
Ni4	0.51121(3)	0.81780(3)	¾	0.0113(1)	1
Ni5	0.53804(3)	0.99490(3)	¾	0.0112(1)	1
Ni6	0.24017(3)	0.04828(3)	¾	0.0158(1)	1
Ni7	0.31193(3)	0.03169(3)	¼	0.0113(1)	1
Ni8	0.33691(3)	0.20830(3)	¼	0.0127(1)	1
Ni9	0.38823(3)	0.02110(3)	¾	0.0115(1)	1
Ni10	0	0	¾	0.036(3)	0.237(9)
Ni11	0.9596(7)	0.0037(6)	¾	0.080(4)	0.12
Si1	0.27083(7)	0.09899(7)	¼	0.0157(3)	1
Si2	0.27995(6)	0.97990(6)	¾	0.0104(2)	1
Si3	0.57934(6)	0.92759(6)	¾	0.0093(2)	1
Si4	0.42947(6)	0.95378(6)	¾	0.0097(2)	1
Si5	0.55198(6)	0.74920(6)	¾	0.0088(2)	1
Si6	0.40148(6)	0.77574(6)	¾	0.0088(2)	1

Table S5. Atomic coordinates and equivalent thermal displacement parameters for n = 6.

Site	*x*	*y*	*z*	U_{eq} /Å^2	SOF
La1	0.34983(2)	0.89041(2)	¾	0.00866(6)	1
La2	0.21986(2)	0.74057(2)	¾	0.00968(6)	1
La3	0.49976(2)	0.08993(2)	¼	0.00863(6)	1
La4	0.07054(2)	0.72076(2)	¾	0.00875(6)	1
La5	0.19973(2)	0.87073(2)	¾	0.00930(6)	1
La6	0.19782(2)	0.04862(2)	¼	0.01261(7)	1
La7	0.37012(2)	0.76023(2)	¾	0.00970(6)	1
Ni1	0.0848(6)	0.9383(7)	0.207(5)	0.023(3)	0.14(2)
La1A	0.0681(9)	0.9816(5)	0.664(4)	0.024(1)	0.092(3)
Ni1B	0.9581(7)	0.9951(5)	¾	0.088(6)	0.118(5)
Ni0B	0	0	0	0.034(3)	0.118(4)
Si1B	0.0926(5)	0.9873(3)	¾	0.037(2)	0.56(2)
Ni1B	0.0969(4)	0.9531(5)	¼	0.015(1)	0.52(3)
Ni0A	0	0	¾	0.034(3)	0.187(7)
La0A	0.0634(5)	0.9828(3)	¾	0.024(1)	0.276(9)
Ni1	0.40770(2)	0.85559(2)	¼	0.0112(1)	1
Ni2	0.42727(2)	0.72350(2)	¼	0.0114(1)	1
Ni3	0.38808(2)	0.98666(2)	¼	0.0114(1)	1
Ni4	0.10516(2)	0.81613(2)	¼	0.0125(1)	1
Ni5	0.23744(2)	0.96693(2)	¼	0.0116(1)	1
Ni6	0.31151(2)	0.97444(2)	¾	0.0118(1)	1
Ni7	0.16205(3)	0.95415(2)	¾	0.0162(1)	1
Ni8	0.44181(2)	0.12502(2)	¾	0.0111(1)	1
Ni9	0.25705(2)	0.83579(2)	¼	0.0111(1)	1
Ni10	0.53818(2)	0.00626(2)	¼	0.0113(1)	1
Si1	⅓	⅔	¼	0.0095(4)	1
Si2	0.31356(5)	0.79761(5)	¼	0.0095(3)	1
Si3	0.46383(5)	0.81722(5)	¼	0.0092(3)	1
Si4	0.16271(5)	0.77795(5)	¼	0.0091(2)	1
Si5	0.44465(5)	0.94981(5)	¼	0.0092(2)	1
Si6	0.29446(5)	0.93028(5)	¼	0.0097(2)	1
Si7	0.40493(5)	0.03054(5)	¾	0.0105(2)	1
Si8	0.25525(5)	0.01149(5)	¾	0.0112(2)	1
Si9	0.14396(5)	0.91050(6)	¼	0.0153(3)	1
